# Improved GNSS Localization and Byzantine Detection in UAV Swarms

**DOI:** 10.3390/s20247239

**Published:** 2020-12-17

**Authors:** Shlomi Hacohen, Oded Medina, Tal Grinshpoun, Nir Shvalb

**Affiliations:** 1 Department of Mechanical Engineering, Ariel University, Ariel 4070000, Israel; shlomiha@ariel.ac.il (S.H.); odedmed@ariel.ac.il (O.M.); nirsh@ariel.ac.il (N.S.); 2 Department of Industrial Engineering and Management, Ariel University, Ariel 4070000, Israel; 3 Ariel Cyber Innovation Center, Ariel University, Ariel 4070000, Israel

**Keywords:** FANET, UAV swarm, GNSS localization, Byzantine detection, pool testing

## Abstract

Many tasks performed by swarms of unmanned aerial vehicles require localization. In many cases, the sensors that take part in the localization process suffer from inherent measurement errors. This problem is amplified when disruptions are added, either endogenously through Byzantine failures of agents within the swarm, or exogenously by some external source, such as a GNSS jammer. In this paper, we first introduce an improved localization method based on distance observation. Then, we devise schemes for detecting Byzantine agents, in scenarios of endogenous disruptions, and for detecting a disrupted area, in case the source of the problem is exogenous. Finally, we apply pool testing techniques to reduce the communication traffic and the computation time of our schemes. The optimal pool size should be chosen carefully, as very small or very large pools may impair the ability to identify the source/s of disruption. A set of simulated experiments demonstrates the effectiveness of our proposed methods, which enable reliable error estimation even amid disruptions. This work is the first, to the best of our knowledge, that embeds identification of endogenous and exogenous disruptions into the localization process.

## 1. Introduction

Swarms in nature perform tasks that are beyond the capabilities of an individual agent. This phenomenon motivates research in the field of robotic swarms. Among the tasks investigated are mapping [1,2], improving crop quality in agriculture [3], manipulating loads in dense workspaces [4], and searching tasks [5]. *Localization* is usually assumed for all tasks (e.g., [6]). The term localization here refers to either (1) absolute localization of each of the swarm agents; or (2) the *swarm’s distribution*, i.e., relative localization of each of the swarm agents regarding the swarm’s center.

One way of obtaining localization was applied in [7] each agent generates chirps in a predefined rate and frequency. The sound waves are picked up by an on-board microphone array and the data is used to estimate the target’s direction.

*Unmanned aerial vehicle* (UAV) is a predominant type of agents investigated. UAVs exemplify a wide range of configurations. Their flight-range and maximum flight-altitudes vary extensively, as well as their sizes, which range from a few centimeters to dozens of meters [8].

A swarm of UAVs can be harnessed to form a *flying ad-hoc network* (FANET) [9], aimed to provide an accurate spatial position of the swarm throughout a given mission (other purposes are out of the scope of this paper). Reliable measurement and transmission are hence critical for missions such as search and rescue applications and military uses [10,11,12], and even for parcel delivery [13].

For most civilian purposes such a swarm should operate in urban areas (or even indoors [2]) where localization of global navigation satellite systems commonly experiences multipath effect and non-line-of-sight reception [14]. These cases are known to generate up to 50 meters of positioning error, which makes it hard even to predict due to multipath complexity [13].

One way to handle the positioning error is to fuse sensory data by incorporating complementary sensors: usage of inertial measurement units [15]; implementation of light detection and ranging scanners to obtain localization via simultaneous *localization and mapping* technology [16,17]; use of a camera-based system endowed with an optical flow algorithm [18]; and incorporation of a similar strategy in which stereo vision systems are mounted on the UAVs [19].

Table 1 provides a bird’s-eye view of the latest research on the subject and their characteristics. For example, Amer et al. [20] presented a method to overcome the pitfalls in urban drone localization, which can be used in additional to the GNSS data. A deep convolutional neural network computes the unique characterization of the urban area to identify the drone’s position. the study of deMiguel et al. [21] introduced a method to improve localization of autonomous vehicles by using LiDAR. By applying Monte Carlo localization approach, they were able to improve the localization calculation in difficult scenarios for GNSS, such as urban canyons, and to overcome the kidnapped-robot-problem that commonly arises in probabilistic localization methods. Goel [22] developed a swarm localization system using GNSS, IMU sensors, and ultra-wideband sensors. His results show that it is possible to achieve accuracy of about 4m in a cooperative swarm by dismissing GNSS and using only low-cost sensors. All the above methods are vulnerable to erroneous measurements that can be a result of adversary agents, malfunctions, and geographic zones where GNSS reading is of low credibility.

Another approach is to use supplementary “chief” agents [23], or some better positioned agent [24,25]. The main problem with such solutions is that they rely on some centralized entity that constitutes a single point of failure.

The term *Byzantine failure* [26], used in *network security theory*, corresponds to a communicating node (agent) that continues operating, but does so improperly. The improper operation can be due to hardware errors, software bugs or malice, such as being infected by a virus. A Byzantine failure may result in delivery of false information, flooding of the network with garbage traffic, or corruption of other agents’ communication packets. Hereinafter, we focus our attention on false information, since the computation of localization information highly depends on accurate inputs from all involved agents. Some TCP/IP protocols do not provide authenticating mechanisms and are thus vulnerable to spoofing attacks (see, for example, [27]); a spoofing attack in our context makes an agent conclude it is located somewhere other than its actual location which falls into the problem in hand as well.

A common approach of dealing with Byzantine failures (e.g., caused by spoofing, malfunction, noise) is to detect the problematic agents and isolate them, e.g., in our case disregard their inputs in the localization computation. To this end, methods for anomaly or outlier detection [28,29] may be applied. Also, model fitting techniques, such as RANSAC [30], can be adapted for purposes of outlier detection [31]. However, outlier techniques that target general data turn out to be unfitting for the problem at hand. This is because of the nature of GNSS localization data—every possible piece of data, whether it is accurate or faulty, may result from a perfectly functioning agent due to the inherently noisy data in this domain.

Although excessive noisiness is a hindrance to detection methods, another feature of GNSS data serves as an enabler for detection. Localization information is being continuously exchanged between agents; thus, accumulative effects may be potentially observed. A method for autonomous GPS satellite failure detection is provided by Parkinson and Axelrad [32]. They define the range residual parameter, which depends on the difference between the measured pseudorange and the range computed based on the estimates of position and clock offsets. Then, to detect faulty satellites, they compare the statistics of this measured parameter to the theoretical expected statistics. A somewhat similar approach to the one taken here is given by Walter and Enge [33]. A least-squares solution is first computed from the pseudorange of a multitude of satellites. Their conditions cannot be met exactly; thus, the researchers quantify the compatibility of the observation, to evaluate the goodness of the fit. Leonardi and Gerardi [27] use airplane/transmitter RF-level features to detect false massages. They compare newly coming massages to an estimated signature of a legitimate message by using Kolmogorov–Smirnov test, which we apply here as well in a somewhat different manner.

The extraction of information regarding an agent’s “honesty” or “effectivity” in distributed and dynamic environments is commonly achieved by *reputation systems* [34,35]. However, general reputation systems, such as Beta [36] and EigenTrust [37], cannot be applied herein since the mere reputability of a single GNSS piece of data can only be derived as part of the localization computation. Actually, domain-specific reputation systems for UAVs and FANETs do exist [38,39]. However, none of them deal with localization computation process at hand. Consequently, one must develop designated solutions that relate to localization data and can identify ongoing trends of Byzantine behavior.

**Figure 1:**
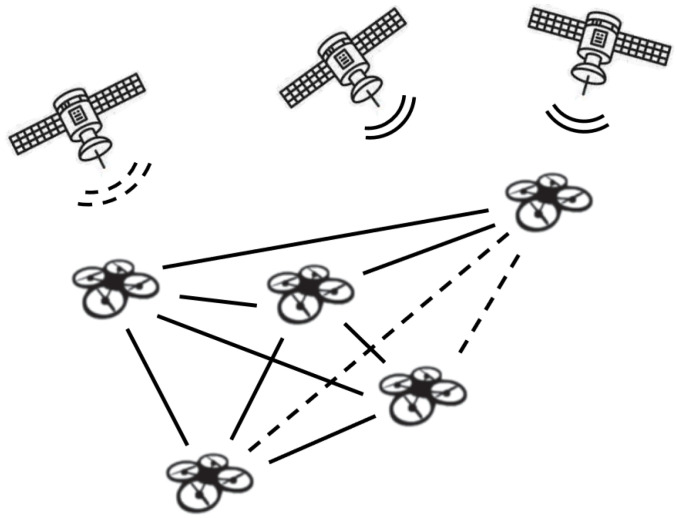
A UAV swarm in which all agents communicate their estimated locations and confidence to all other swarm members. A GNSS reading is available for all. In the endogenous problem, some agents communicate faulty information (marked in dashed lines). The Byzantine information may be communicated only occasionally to some of the agents. In the exogenous problem (e.g., signal reflection problems or jamming) the GNSS signal in a certain geographical area cannot be trusted (dashed node).

Indeed, *endogenous* problems to the swarm caused by Byzantine agents within the network can lead to GNSS disruption. Nonetheless, *exogenous* problems, such as geographic zones with GNSS interference or jamming/blocking [40], are also a major source of concern. The two types of problems are illustrated in Figure 1. In this paper, we tackle both problem types.

### 1.1. Contribution and Paper Organization

In prior work [41] the authors introduced an algorithm for estimating a swarm distribution. The algorithm is based on a simple sensing capability of measuring an angular location of other agents (relative to a global *x*-axis). Alternatively, one may measure the mutual distances between swarm members by using low-weight range sensors. the swarm distribution is thus obtained using only on-board sensors, without any external positioning systems. All agents share their measurements at each timeframe and an *Extended Kalman filter* (EKF) is applied for estimating the swarm distribution.

In this paper, we introduce a method borrowed from recent advances in COVID-19 epidemiology called *test pooling*. Pool testing, first studied by Dorfman [42], is a generic name for procedures that identify certain objects by testing groups of items, rather than individual ones and singling out an object by intersecting the identified groups. We show how this method provides information as to which of the agents transmit erroneous measurements [43]. Furthermore, the algorithm can indicate geographic zones where GNSS reading is of low confidence. By doing so, the algorithm can handle both endogenous and exogenous GNSS disruption scenarios. We exemplify how applying such a strategy for a swarm with more than 15 agents completely overcomes measurements problems.

This paper is organized as follows: Section 2 presents the improved localization main formulation and algorithm for the stochastic case. Section 3 presents the case where a fixed number of Byzantine agents impairs the localization process and should be detected. Section 4 deals with the case of a varying number of Byzantine agents. A solution for locating a Byzantine agent by using pooled sample testing is discussed in Section 5. In Section 6 we study the localization with exogenous disrupted signal. Finally, we conclude the paper in Section 7 and share our plans for future research.

### 1.2. Nomenclature

Throughout this paper A will denote the set {1,…,*n*} of all agents in the swarm. A hat decoration $\hat{\zeta }$ is used to indicate an estimation of a quantity ζ; the time frame *k* is indicated by a subscript and the *i*th agent-related quantity i∈A is given by a superscript $\zeta_{k}^{i}$ Locations and measurements are marked by ***x*** and ***z***, respectively. We use N (*μ*,Σ) to notate a Gaussian distribution with an expected value *μ* and a covariance matrix Σ.

## 2. Improved Localization—The Case of No Disruptions

Each agent is assumed to be equipped with a GNSS receiver and an inertial measurement unit (IMU). In addition, an on-board sensory system enables measuring the distances to other agents.

The *distance measurement* of agent *i* regarding to some another agent j∈A\i at time-step *k* is denoted by $z_{k}^{i,j}$ (aka an *observation*). As for now, we assume that at each time-step *k*, all agents i∈A communicate only their *estimated locations* $\left\{{\hat{x}}_{k}^{i}\right\}$ and the confidence levels, i.e., the corresponding standard deviations (STD), which will be discussed later in this section. the state model we shall use is a discrete-time location equation (written for agent *i*):

(1) $$x_{k}^{i}=x_{k-1}^{i}+d_{k}^{i}+\nu_{k}^{i}$$

where $x_{k}^{i}$ marks *location*, $d_{k}^{i}$ is the instantaneous step vector and $\nu_{k}^{i}∼N\left(0,\Sigma_{d}^{i}\right)$ is the process noise; $\Sigma_{d}^{i}$ indicates a symmetric positive-definite 3×3 square matrix (or 2×2 in case of a two-dimensional problem) which is assumed to be known. the distance measured between agent *i* located at $x_{k}^{i}$ and some other agent *j* located at $x_{k}^{j}$ is given by:

(2) $$z_{k}^{i,j}=∥x_{k}^{i}-x_{k}^{j}∥+\omega_{k}^{i,j}$$

where $\omega_{k}^{i,j}∼N\left(0,\sigma^{i,j}\right)$ stands for the unbiased measurement noise. We denote the *measurements vector* as a concatenation of all measured distances.

(3) $$z_{k}^{i}=f\left(x_{k}^{i}\right)=:{\left\{z_{k}^{i,j}\right\}}_{j\in A\backslash i}$$

### 2.1. Improved Localization Scheme Based on Distance Observations

As commonly the case in Bayesian processes, the localization process herein is comprised of two main phases—the *prediction* phase and the *measurements update* phase. Many schemes for GNSS-based localization consist of odometry measurements in the prediction phase, followed by a measurement update phase that relies on GNSS pseudo-ranges and Doppler observations. In the proposed scheme, the measurement update is performed twice: first by the GNSS measurements and then using the distance measurements. To apply the second measurement update, other agents’ estimated locations are considered to be benchmarks. Thus, since agents’ estimated locations are random variables, location uncertainty should be added to the inherent sensor uncertainty (denoted $\sigma_{d}$).

In the measurements update phase the state innovation is computed as the difference between the actual measurement $z_{k}^{i,j}$, and the predicted one $f({\hat{x}}_{k}^{i},{\hat{x}}_{k}^{j})$. The computed distance (given in Equation (2)) involves the agents’ location uncertainties. Considering the estimation-error covariance, at each time-step *k*, each agent *i* uses an EKF to calculate the best available prediction for this uncertainty denoted by $P_{k}^{i}$. Following [44], we convolve the location distribution of each of the two agents *i* and *j*; we then take the confidence level to be the maximal eigenvalue of the prediction. Please note that the covariance matrix of a convolution of two normal distributions is simply the sum of the two covariance matrices. Finally, we transverse the computed distance uncertainty to the sensor’s measurement error STD. Therefore, the approximated STD of the measured distance between agents *i* and *j* can be taken as:

(4) $$\sigma^{i,j}=\sigma_{d}+\lambda_{max}\left(P_{k}^{i}+P_{k}^{j}\right)$$

where $\lambda_{max}\left(A\right)$ is the maximal eigenvalue of the matrix *A*. Since $\lambda_{max}\left(A+B\right)\leq \lambda_{max}\left(A\right)+\lambda_{max}\left(B\right)$, the agents may share only the maximal eigenvalue of their covariance matrices. In turn, the confidence level calculation takes their summation as an upper bound.

The improved localization scheme is provided in Algorithm 1 (for a more thorough discussion the reader is referred to ([45] §7)). We mark the GNSS observation function by $\rho_{k}=h\left(x_{k}^{i}\right)$ and its Jacobian by $H_{k}^{i}=\frac{\partial h}{\partial x}{|}_{{\hat{x}}_{k}^{i}}$. We further denote the distance observation function by $f(x_{k}^{i})$ (see Equation (3)) and its Jacobian by $F_{k}^{i}=\frac{\partial f}{\partial x}{|}_{{\hat{x}}_{k}^{i}}$. Please note that the *observation model matrix* $F_{k}^{i}$ of the update phase is provided by the Jacobian matrix of the measurement function:

(5) $$F_{k}^{i}={\left[\ldots ,\frac{{\hat{x}}_{k}^{i}-{\hat{x}}_{k}^{j}}{∥{\hat{x}}_{k}^{i}-{\hat{x}}_{k}^{j}∥},\ldots \right]}_{3\times ∥A∥-1},\;\;j\in A\backslash i$$

The uncertainty of agents’ locations is taken as the *observation noise*. Thus, the covariance of the *observation noise matrix* $R_{k}^{i}$ is assumed to be the following diagonal matrix, with its columns corresponding to all j∈A\i:

(6) $$R_{k}^{i}={\left[\begin{array}{ccc} \sigma^{i,1} & & 0\\ & \ddots & \\ 0 & & \sigma^{i,n} \end{array}\right]}_{n-1\times n-1}$$

**Algorithm 1:** EKF-based improved localization
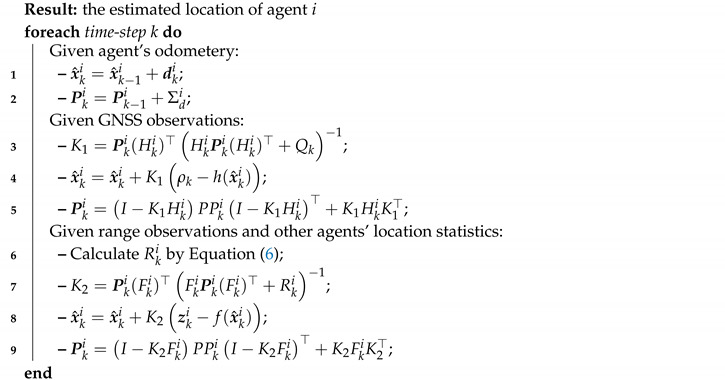


### 2.2. Simulation Results

To demonstrate the efficiency of the localization process, a simulated experiment was implemented. Our experiments were run on a hardware comprised of an Intel i7 processor and 8GB memory. The simulation infrastructure consists of a workspace scene of size 400 m × 400 m with *n* agents translating in random directions. Obviously, the computational rate should comply with the agents’ velocities. Here, we assumed that the agents’ velocity is 2 m/s as for most hovering drones (e.g., DJI Mavic), while the calculation rate is 2 Hz.

Each agent is equipped with a sensor that measures the agent’s distance from other agents (such a sensor may be in practice a LoRa sensor, which is based on a spread spectrum modulation). We assume that the sensors cover the entire workspace. the measurements’ noise corresponds to the error in distance simulated as $\sigma_{d}=2$ m; the length of the step size in each time-step is simulated as a normal distribution vector with variance of 1m; the odometry noise is taken as 0.7 m. Finally, the GNSS observations applied localization with normally distributed noise with zero mean and STD of 30 m; a distorted GNSS receiver applies the localization with a uniform noise of ±15 m that is added to the above normally distributed noise. To illuminate the performance of the proposed scheme, the noises in our simulations were set to be greater than those of common GNSS receivers (e.g., [46]) and odometry devices (e.g., [47]). This simulation setup was used for all the experiments throughout the paper. In addition, for Section 2, Section 3, Section 4 and Section 5 the agents follow random paths with the additional non-collision constraints. In Section 6 we use an artificial potential field method for controlling the agents’ movements to cover the geographical landscape, i.e., the resultant artificial force attracts the agents to areas not yet visited, to avoid local minima we apply constant perturbation to the algorithm (cf. [48]).

**Figure 2:**
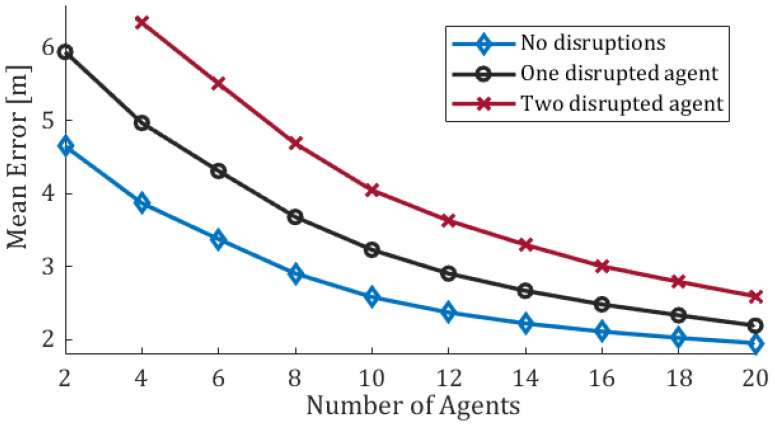
The number of agents in the swarm vs. The mean estimation-error throughout the entire run for different number of disrupted agents. Each run lasted 300 time-steps and each data point represents the mean of 100 runs; the raw localization error is assumed to be 30 m.

Initial locations of the agents as well as their estimated states are chosen randomly. Following that, a simple Kalman filter is applied to estimate the agents’ positions. Convergence results for different swarm sizes (2 to 20 agents) and different number of disrupted agents (0 to 2) are depicted in Figure 2. Each data point represents the mean of 100 simulation runs of 300 time-steps each.

Consider first the case of no disruptions (light blue line), which is the focus of this section. As expected, at some point (n>12 agents) the addition of more information to the EKF scheme leads to only marginal improvement in performance. The results show that the principle of *diminishing returns* holds here as well (for example, see [49]). The upside of this phenomenon is that when the swarm is large enough, the absence of distance observations from some of the agents (e.g., due to communication failures) does not significantly impair the quality of localization. This can be thought of as a *robustness* feature of the scheme.

**Figure 3:**
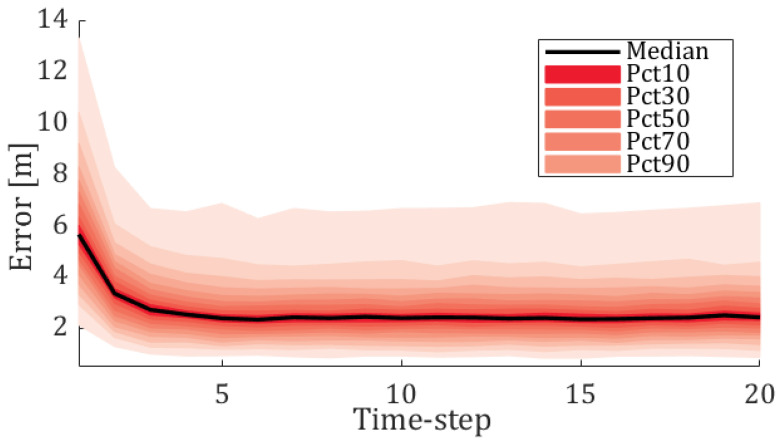
Fanplot of the algorithm’s performance for n=16 agents over time-steps. The middle solid line indicates the median estimated error measured in meters. The shaded bands present the percentages of position error at each time-step. The performances are statistics of 100 Monte Carlo runs.

To obtain further insights regarding the performance of the localization scheme, we consider a more specific setting in which we fix some parameters to enable illumination of others. Figure 3 shows the simulation results on the same infrastructure but for swarms of a fixed size of n=16 agents, of which a single agent is disruptive. This experiment focuses on two aspects—convergence speed of the localization scheme and the error distribution among the agents.

As can be clearly observed from Figure 3, the convergence of the localization scheme is very fast (∼5 time-steps). Regarding the estimation error, here it is presented in percentiles. The reported results indicate that for most of the agents the errors are below 3 m; the 90th percentile is about 7 m, i.e., for 90% of the agents the error is smaller than 7 m. Please note that relying solely on GNSS observations (without exchanging distance observations) leads to a mean error of 8.74 m on a similar setting, albeit without disruptions. The latter datum confirms the importance of distance observations to the accuracy of the localization scheme.

Figure 2 also presents results in the presence of one or two disruptive agents. The black and red lines show the mean error of the remaining “normal” agents (i.e., excluding the error of the disruptive agents). Nonetheless, and even when considering disruptions by just one or two agents, the effect of disruption on the quality of localization is clearly substantial. This motivates our disruption-detecting algorithms that are presented in the following sections.

## 3. Identifying Disruptions—The Case of a Fixed Number of Byzantine Agents

As shown in Figure 2 in the previous section, the presence of disruptive (Byzantine) agents considerably impairs the accuracy of the localization process. To deal with such disruptions we adopt a strategy of explicitly detecting the Byzantine agents. Following that, the other agents can disregard the measurements received from the pinpointed agents, hence achieve successful localization. Such a strategy has been successfully applied in the closely related domain of wireless ad-hoc networks [50].

For simplicity of presentation, we focus herein on the case of a single Byzantine agent. Nonetheless, the scheme discussed in this section can be naturally adopted to deal with any number of Byzantine agents, as long as this number is fixed and known a priory. Alternative schemes should be used in case the number of Byzantine agents is unknown or dynamic; such schemes are discussed in the subsequent sections.

### 3.1. Byzantine Detection Scheme

Our basic Byzantine detection scheme is based on *likelihood* values that are gathered by all the agents. Each agent considers a peer with minimal likelihood as a suspect. Additionally, the scheme “remembers” suspicions from the last τ time-steps. Thus, together with the current time-step, a suspects list *S* of τ+1 time-steps is maintained. Please note that such an algorithm does not depend on the type of false information a Byzantine agent communicates (i.e., a correlated information bias or a noisy one).

Algorithm 2 presents the pseudo-code of the basic Byzantine detection scheme. At every time-step *k*, each agent i∈A retrieves its own estimated location (Line 1), and subsequently calculates a set of likelihood values for the other agents j∈A\i (Line 2). This calculation is obtained by:

(7) $$L_{k}^{i,j}\left({\hat{x}}_{k}^{j}|z_{k}^{i,j}\right)=\exp\left(-\frac{1}{2}{\left(∥{\hat{x}}_{k}^{i}-{\hat{x}}_{k}^{j}∥-z_{k}^{i,j}\right)}^{2}\right)$$

where $L_{k}^{i,j}$ is the *likelihood* value of agent *j* as calculated by agent *i* in time-step *k*. Next, each agent *i* reports the index $j_{k}^{i}$ of the agent he suspects, i.e., the one with the minimal $L_{k}^{i,j}$ value (Line 3).
**Algorithm 2:** Detect a Byzantine agent
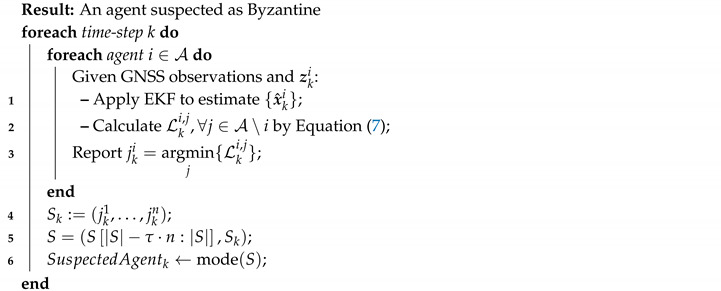


The list of reported suspects in time-step *k* is denoted $S_{K}$ (Line 4). A second list *S* is then updated. This list constitutes a moving time-window of τ+1 time-steps of suspects, is constructed in Line 5. Finally, in Line 6, $SuspectedAgent_{k}$ (the suspected agent in time-step *k*) is taken as the statistical mode of *S*, i.e., the agent that most frequently appears in *S*.

Algorithm 2 can be implemented in two variants—*centralized* and *distributed*. In the centralized variant, each UAV agent reports $j_{k}^{i}$ to some central unit; next, the central unit gathers the information and obtains $SuspectedAgent_{k}$; finally, it reports $SuspectedAgent_{k}$ to the UAV agents. Alternatively, in the distributed variant the UAV agents report their $j_{k}^{i}$ suspicions to all their peers; then, the computation of the suspected agent is conducted independently on board each UAV.

There is a clear tradeoff between the two variants. The centralized variant is more economical in terms of network load, especially when the number of agents is large. However, the centralized variant requires a central unit, which is either an additional agent or one of the UAVs. In any case, the central unit constitutes a single point of failure, which is the Achilles heel of many existing solutions, e.g., [23,24,25]. Such problems are inherently solved with the use of the distributed variant at the price of more extensive messaging. Here, we implemented only the centralized variant.

### 3.2. Simulation Results

**Figure 4:**
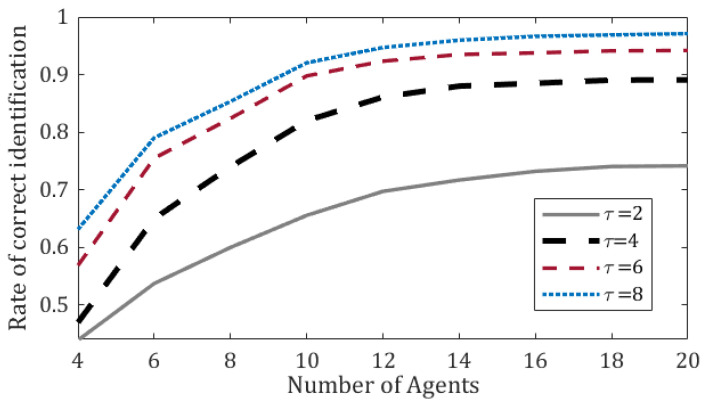
Rate of correct identification of a single fixed Byzantine agent by Algorithm 2 as a function of the number of agents. Different values of the τ parameter are examined. Each graph point displays the mean of 100 runs.

The simulation herein focuses on the scenario of a single Byzantine agent; the considered Byzantine effect is *static*, i.e., the identity of the Byzantine agent does not change throughout the course of a single simulation run. We follow a similar simulation infrastructure to that of the previous section, but this time we also apply Algorithm 2 to identify the Byzantine agent. Figure 4 presents the rate of correct identification as a function of the swarm size for various τ values.

As depicted in Figure 4, longer time-windows (higher values of τ) improve the rate of correct identification with window-length being very short in terms of absolute time. This was expected since the Byzantine agent is fixed in the examined scenario; thus, a longer time-window translates to a larger amount of *relevant* data that contributes to the accuracy of Algorithm 2. However, in case the Byzantine agent is not fixed, perhaps shorter time-windows would be more suitable to accommodate such dynamic behavior. Another potential form of dynamicity is when the number of Byzantine agents changes or is just unknown upfront. In the next section we propose a more appropriate method for handling such scenarios.

## 4. Identifying Disruptions—The Case of a Varying Number of Byzantine Agents

Consider the case in which the number of Byzantine agents is not known upfront. In such a case, Algorithm 2 may fail because it reports a single suspected agent (or if a minor change is adopted, a fixed-size list of suspected agents) at every time-step. This limitation can be waived by applying a comparison of the sets of agents’ likelihoods. To this end, we implement the *Kolmogorov–Smirnov* test (K–S) [51], which is discussed next.

### 4.1. Byzantine Detection by the Kolmogorov–Smirnov Test

To compare two continuous one-dimensional probability distributions of sizes *n* and *m*, it is common to use the K-S test.

(8) $$KS:=\underset{j}{\sup}∥\Lambda_{j}-\Omega_{j}∥$$

where the null hypothesis is rejected with probability α if $KS>c\left(\alpha \right)\sqrt{\frac{n+m}{n\cdot m}}$; c(α) is broadly explained in [52].

Here, we test all agents iteratively, by comparing two sets of reported likelihoods. For the test of the agent *j*, the first set is marked by $\Lambda_{j}$, which includes all reported likelihoods arrived from the whole swarm, except for the likelihoods related to agent *j*, i.e., we examine how well the swarm does without considering the data provided by agent *j*. The second set $\Omega_{j}$ includes only the likelihoods related to agent *j*. In other words, we use the K-S test to compare the sets of observation likelihoods with and without agent *j*. Algorithm 3 presents the pseudo-code of the scheme.

### 4.2. Simulation Results

**Figure 5:**
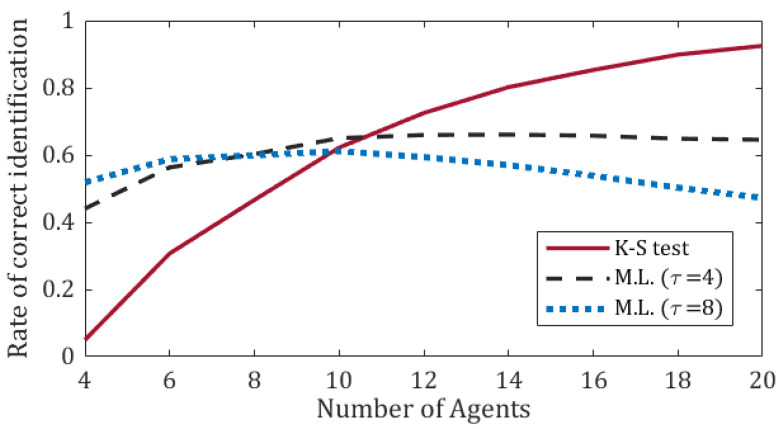
Comparison of correct Byzantine agent identification rates between maximum likelihood and K–S test. The identity of the Byzantine agent changes in each time-step with 10% probability.

Figure 5 presents the correct identification rate for a scenario of a single Byzantine agent with changing identity. The identity of the Byzantine agent changes in each time-step with 10% probability. The aim of this experiment is to investigate a *dynamic* Byzantine effect. Thus, we use the same setting as in Figure 4, except for the identity of the Byzantine agent, which now changes dynamically.

In this experiment we compared a *maximum likelihood test* (ML) with the K-S test. The performance of ML are presented for τ=4 and τ=8. The results indicate that for small swarms (n<10) ML provides better results. This is attributed to the fact that small swarms have small sets (Λ,Ω) to compare, for which the K-S test does not perform well. However, the effectiveness of the K-S test improves with the increase of the swarm size.
**Algorithm 3:** Detect Byzantine agents using K-S test
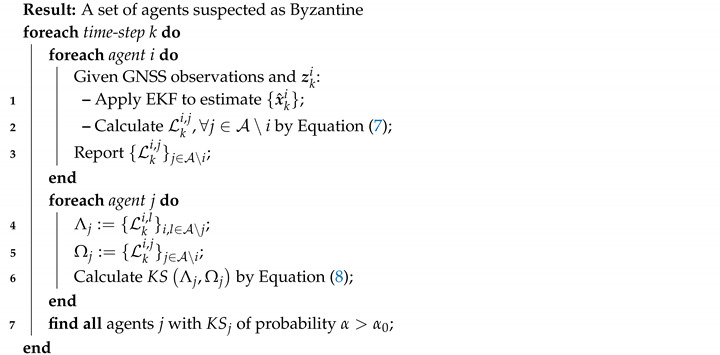


## 5. Reducing Communication Traffic and Computational Effort by Pooled Testing

This research was conducted during the COVID-19 virus pandemic. In absence of a vaccine or medicine drugs for the COVID-19, it turned out that it is vital to have as many and as fast virus-infection tests as possible, to detect early outbreaks of the infection. This traditional approach was found to be limited due to insufficient resources. To speed up the testing, attempts were made to use swabs of multiple patients grouped together and tested, and to recover the positives by elimination [53,54]. We adopt these methods herein to recover multiple Byzantine agents.

Consider the case where it is known that a single agent out of 25 is Byzantine. The naive way of recovering it is by applying a scheme in which the swarm excludes a single agent at a time from the localization. For each such exclusion an estimation is made as to the closeness of the localization to the GNSS readings. The problem is that such mode of operation requires 25 steps. Moreover, for the case of non-constant Byzantine communication this will not do.

A shorter alternative is to apply the *bisection method*, i.e., to eliminate 12 agents following the first test, then 6, and so on. This process will be concluded in up to 5 steps.

**Naive pooling:** Our experiments (see Section 6.2) indicate that there is an optimal pool size for detecting geographical disrupted areas. Considering this, one can use pool testing for Byzantine agents by testing multiple agents at once. If the result is negative then all the agents in the group are cleared. For example, suppose 25 agents should be tested in pools of up to 6 agents. In pool testing, a random pool of 5 agents is selected and Algorithm 3 is applied on it. The suspected agents of the current pool are then reported. It should be noted that if there are too many Byzantine agents in a given pool then the initial location estimation of the agents would be noisy; in such a case, this strategy would not suffice. To overcome this shortcoming, the pools need to be repeatedly and randomly selected each time.

Consider the case in which 12 pools of 8 agents each are examined. Assuming that 3 positive tests are found in different pools, then 12+8×3=36 tests will be required overall. Therefore, the choice of pool size can be optimized depending on the prevalence.

Yet another consideration one should take into account is the range of appropriate group sizes for the swarm localization. Too large groups may result in averaging out the effect of the Byzantine agent, whereas very small groups may turn out to be too noisy.

**Optimal replication pooling:** Another method that can be applied relies on replications [53]. In this method, each agent is allocated in the first pool that has a vacancy. A replica of that same agent is then allocated in the first pool with the smallest number of associated agents, while making sure that an agent and its replica are included in two different pools. Additionally, the intersection of any pair of pools corresponds to a single agent.

**Figure 6:**
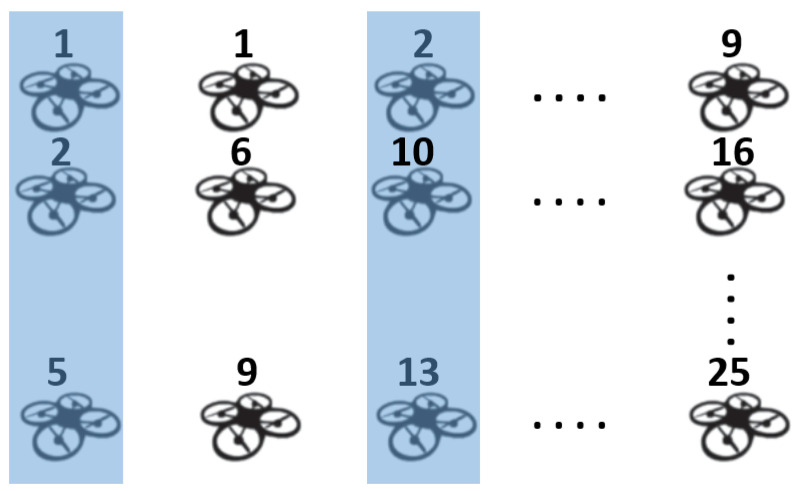
Optimal replication pooling for 25 agents. Each column represents a pooled test. The marked columns correspond to agent 2.

For example, 25 agents may be allocated to 10 pools of 5 agents each. Figure 6 illustrates such a pool ordering. In this example, 10 tests are required to recover a single Byzantine agent. If more than two pools are detected as problematic, then there is definitely more than a single agent suspected as Byzantine. For instance, if 4 pools are found problematic, then there can be up to 6 suspected agents. In such cases we follow [53] and separately check all the 6 agents.

Regardless of the chosen pooling technique, both the communication traffic and the computational effort significantly reduce due to two reasons. First, pooling leads to an overall lower number of tests. Second, each test is of smaller size because it is conducted only among the members of the pool, and not by the entire swarm.

Recall that Byzantine agents are recovered using K-S tests on the sets Λ and Ω. In the schemes described in this section, the latter set corresponds to a set of agents which are included in the pool. Thus, the K-S test is less reliable in this case. Nonetheless, we shall exemplify the pooling schemes in scenarios that include exogenous disrupted signals, which are discussed next.

## 6. Identifying Exogenous Disruptions

Consider the case in which there is a certain area where signal is disrupted by some exogenous source, i.e., not by agents within the swarm. The best strategy to avoid such disturbances would be to recover the problematic geographical area and to ignore agents that are currently there.

### 6.1. Localization with Exogenously Disrupted Signals

For simplicity of discussion, let us first assume that there are some geographical areas where the positioning signal cannot be trusted (without any assumption as to their nature). For example, an area with a jammer in its center may lead to GNSS signal problems for agents passing through the area.

We apply the localization scheme and the K-S test for detecting the disrupted agents as described in the previous sections. To retrieve these areas, we first divide the workspace into an array of grid cells $\Gamma_{q,r}$, which will be the data basis to accumulate the suspicions about disrupted positions.

On the one hand, a suspicious agent (according to the discussed scheme) located in a certain grid cell will increase the confidence of that grid cell being the source of disruption. On the other hand, an agent that is located in a grid cell and is not found to be suspected as having signal distortion will decrease the confidence of that grid cell as a source of disruption. Each increase/decrease is of a single unit of suspicion.

We are interested in devising a probability distribution function of the suspicious areas. To this end, we divide the suspicion value of each grid point $\Gamma_{q,r}$ by the current sum of suspicion values of all the grid cells. We consider only non-negative suspicion values to avoid late detection of new problems. Consider a situation in which a previously clear area (i.e., with no disruptions) accumulates a very large negative suspicion value. If a jammer starts operating in this area, it will take a long time until the suspicion values of the relevant grid cells become positive again. Thus, to avoid such scenarios, we set the minimal suspicion value to zero.

### 6.2. Simulation Results

**Figure 7:**
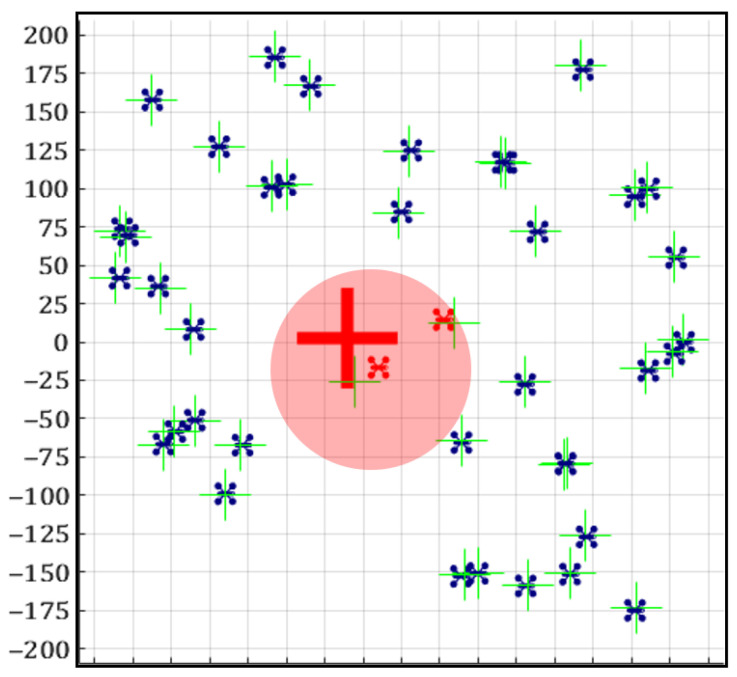
A snapshot of the simulation: the workspace is divided into a 25×25 grid. The dark closed shape corresponds to the area with disrupted GNSS signals; the cross indicates the estimated position of the disrupted area.

We use herein the simulation setup described in Section 2.1. Figure 7 exemplifies the results. the dark circular shape indicates the area in which a noisy GNSS reading is provided (S/N ratio is set to 30 times the ratio in non-disrupted zones). Suspicion values are provided for the cells in the 25×25 grid. The two agents within the disrupted area are found to be suspicious by the K-S test, whereas the other agents are clear of suspicion. Consequently, our scheme identifies the large cross as the center of disruption, which is clearly quite close to the true location of the disruption’s source. Please note that the algorithm does not assume anything about the geometry of the disrupted zone.

**Figure 8:**
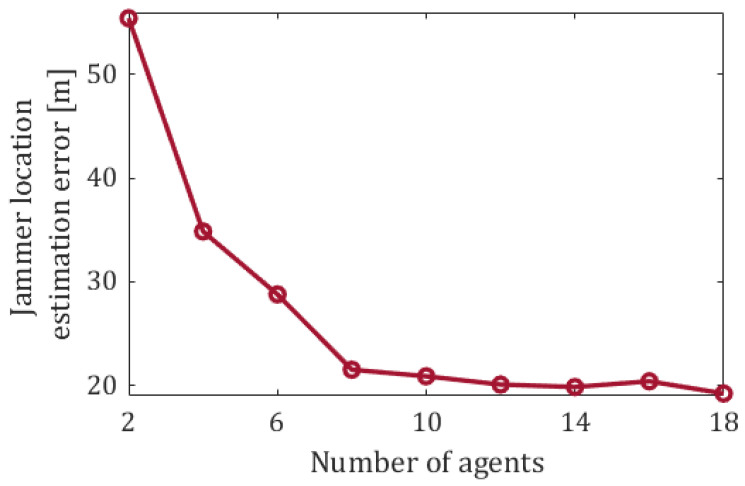
The estimation error of the jammer’s location as a function of the number of agents, with no pooling strategy applied. Each graph point displays the mean of 100 runs.

Obviously, as the number of agents grows so does the rate at which each grid cell is being visited. Figure 8 depicts the jammer’s location estimation error as a function of the swarm’s size; no pooling strategy has been applied in this setting. As expected, the growing rate of cell-visitations increases the accuracy of the location estimation.

**Figure 9:**
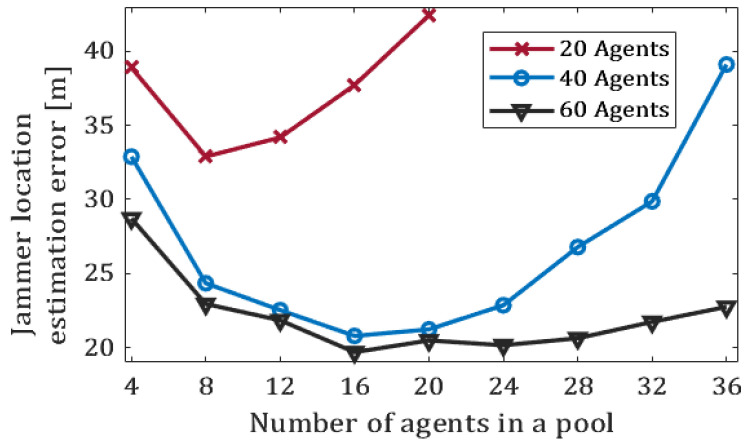
The estimation error of the jammer’s location as a function of the pool size. The simulations consider swarms of 20, 40, and 60 agents; a naive pooling strategy is applied. Each graph point displays the mean of 100 runs.

Next, we examine the use of test pooling in view of exogenous disruptions. Figure 9 displays the effect of the pool size on the accuracy of the location estimation. In this experiment we considered swarms of 20, 40, and 60 agents. A naive pooling strategy was applied. Interestingly, the best estimations of the jammer’s location were obtained when using medium-sized pools (pools of size 8 for 20-agent swarms, and pools of size 16 to swarms of 40 or 60 agents). Conversely, pools of smaller or larger sizes resulted in poorer estimations. We believe that the reason for this is two-fold. When the number of agents in a pool is too small, the benchmark for comparison is not solid enough. On the other hand, when the pool size is chosen to be large, it is more likely that many agents are in the disrupted area, which leads again to a poor benchmark for comparison.

Table 2 concludes the simulation results for different pooling strategies: optimal replication pooling with pool sizes of 5 and 8, naive pooling with pools of size 10, and the case of no pooling. For each of the above strategies, the jammer’s location was estimated in 100 independent runs. The table lists the mean error, the standard deviation over all simulation runs, and the computation time for estimating the jammer’s location. Roughly speaking, as the number of tests decreases, so does the accuracy of estimation. However, at the same time, less tests mean less computational effort and communication overhead. Thus, it is important to understand the trends of each pooling method to choose the most appropriate method for a given scenario. Replication pooling turns out to be very erroneous when applied to small swarms of 20 agents; however, its error drastically decreases when applied on larger swarms. Contrary to that, naive pooling provides a good balance between computation time (it is the fastest) and accuracy (only ’no pooling’ is more accurate) when applied on small swarms; in such settings, using no pooling is about 33% more accurate, but 4 times slower. However, unlike replication pooling and ’no pooling’, the accuracy obtained by the naive pooling method only marginally improves for larger swarms; hence, it seems less fitting for scenarios of large swarms.

## 7. Conclusions

We discussed the localization of a FANET in cases where the GNSS measurements are not satisfactory. The localization was calculated in a two-fold manner: first by the GNSS measurements and then, by applying EKF, we improved the localization of the swarm agents by measuring their relative ranges. In addition, the likelihood of the GNSS observations was used to identify both endogenous (Byzantine agents) and exogenous (geographical areas) disruptions.

According to the experiments on static Byzantine effects, our algorithm works well in identifying a Byzantine agent with efficiency of at least 90% when the swarm size is at least 10 and the time-window is sufficiently long (τ=8 time-steps). As the swarm size increases, more agents participate in the identification process, thus smoothing out the noises. Additionally, more participants in the localization process mean that the benchmark for comparison becomes more solid; consequently, the Byzantine agent is singled out in a clearer manner.

Next, our focus turned to static Byzantine effects. For such scenarios, we used Kolmogorov–Smirnov and maximal likelihood tests to eliminate non-disruptive agents. Furthermore, we showed how such a strategy can be further extended to recover geographic areas where GNSS signal cannot be trusted.

The computational effort required for applying these tests on the entire swarm heavily depends on the size of the swarm; also, the rate at which the tests should be conducted depends on the agility of the agents. Consequently, we incorporated pool testing techniques, which enable efficient recovery of faulty information out of a large data set that otherwise requires immense testing. We implemented two pool testing methods to recover a geographical area where a GNSS jammer is located, and presented the pros and cons of each method. In addition, the size of the pool also plays an important role. In fact, very small or very large pools lead to impaired identification ability, so the optimal pool size should be chosen carefully.

Although there exist many solutions for GNSS localization, as well as many algorithms for detection of Byzantine effects, none of the existing literature combines these two seemingly orthogonal problems. To the best of our knowledge, this work is the first that embeds identification of endogenous and exogenous disruptions into the localization process. We consider this as the key feature of our study, and believe it will pave the way for the development of other solutions of this sort.

In a previous research [55], we implemented a bearing sensor for a simultaneous localization and mapping (SLAM) mission. In future work, we plan to improve SLAM accuracy by incorporating the algorithms presented here. 

## Figures and Tables

**Table 1:** Techniques for improving GNSS. For each research, we mark by ✓ the techniques it incorporates and by ✗ the unsupported techniques.

	Technique					Performances
	IMU Sensor	Range Sensor	Landmarks	Optical Flow	Agent-Agent Interactions	Reported Accuracy
[15]	✓	✗	✓	✗	✗	∼0.05 m
[16]	✗	✗	✓	✗	✗	N.R.
[18]	✗	✗	✗	✓	✗	∼1.5 m
[19]	✓	✓	✗	✗	✗	∼1.5 m
[20]	✗	✗	✓	✗	✗	N.R.
[21]	✓	✓(LiDAR)	✗	✗	✗	<1 m
[22]	✓	✓(LiDAR)	✓	✗	✓	∼4 m

**Table 2:** The performances of various schemes for detecting jammer location in swarms of 20, 40, and 60 agents. The displayed performances are of 100 independent runs per swarm size.

Number of Agents	Pooling Method	Replication Pooling (5)	Replication Pooling (8)	Naive Pooling (10)	No Pooling
20	Mean error [m]	42.844	51.758	21.183	14.288
	STD	16.495	25.530	12.146	10.922
	Mean time [s]	0.0026	0.0022	0.002	0.008
40	Mean error [m]	22.597	28.050	20.981	12.611
	STD	17.846	22.324	12.378	8.903
	Mean time [s]	0.0085	0.009	0.004	0.0135
60	Mean error [m]	21.591	24.759	20.239	9.4872
	STD	17.503	16.813	12.497	5.5110
	Mean time [s]	0.009	0.009	0.009	0.017
